# Research on the Protective Effect of MiR-185-3p Mediated by Huangqin-Tang Decoction (HQT) on the Epithelial Barrier Function of Ulcerative Colitis

**DOI:** 10.1155/2021/4775606

**Published:** 2021-12-21

**Authors:** Zhou Changlin, Zou Ying, Zhang Shuhua, Jiayang Zeng, Honggang Chi

**Affiliations:** ^1^The First Dongguan Affiliated Hospital, Guangdong Medical University, Dongguan, Guangdong 523000, China; ^2^Dongguan People's Hospital, Dongguan, Guangdong 523000, China; ^3^Department of Traditional Chinese Medicine, Dongguan Liaobu Hospital, Dongguan, Guangdong 523000, China; ^4^Department of Traditional Chinese Medicine, The Second Clinical Medical College, Guangdong Medical University, Dongguan, Guangdong 523000, China; ^5^Shunde Hospital of Southern Medical University, The First People's Hospital of Shunde, Foshan, Guangdong 528399, China

## Abstract

**Introduction:**

It has been reported that the traditional Chinese medicine Huangqin-Tang decoction (HQT) has a protective effect on the epithelial barrier function of ulcerative colitis, but its mechanism has not been fully clarified. This study intends to explore the protective mechanism of HQT in regulating microRNA (miRNA) for the first time.

**Methods:**

Based on the Balb/c mice ulcerative colitis model, the mice were given a gavage of 0.1 mL/10 g HQT every day for 7 days; on the 8th day, the colon of the mice was dissected, the length of the colon for the mice was measured, and the score was given based on this. Analysis of colonic mucosal injury was conducted by hematoxylin-eosin staining. Then, the differential miRNA was screened and sequenced in colon tissue using the HiSeq platform. And the differential miR-185-3p gene was verified by RT-PCR. Finally, the effects of HQT on miR-185-3p, occludin protein expression, and transepithelial electrical resistance (TEER) value were observed in combination with the CaCo_2_ intestinal epithelial cell model.

**Results:**

HQT treatment can alleviate the shortening of colon length and reverse the intestinal mucosal injury. miRNA sequencing of colonic tissue showed that miR-185-3p was significantly downregulated in the model group, while HQT could upregulate miR-185-3p, thereby affecting the myosin light chain kinase (MLCK)/myosin light chain phosphorylation (p-MLC) pathway and leading to increased expression of occludin protein, which ultimately protected the intestinal epithelial barrier function.

**Conclusion:**

HQT can protect colon epithelial barrier function by regulating miR-185-3p.

## 1. Introduction

Intestinal mucosal barrier injury is an important cause of ulcerative colitis (UC), while an injured intestinal mucosal barrier can cause increased intestinal permeability, allowing antigens of bacterial or food origin in the intestinal lumen to enter the mucosa, thus triggering an immune response in the intestine and an outbreak of an uncontrollable cascade of inflammatory signals. Studies have shown that increased intestinal mucosal permeability may be an early event in the onset of UC, and patients with US at rest develop increased intestinal permeability and associated intestinal symptoms even though no significant abnormalities are observed under endoscopy [1–3]. Therefore, improving patients' intestinal epithelial barrier function is considered to be the key to effective treatment of UC. The traditional Chinese medicine HQT is a classic prescription for treating dysentery, and it was first recorded in the *Treatise on Febrile Diseases* by Zhang Zhongjing: the combination of sun disease and Shaoyang disease with diarrhea should be treated with Huangqin-Tang decoction. HQT which is composed of Scutellaria, white peony root, date, and roasted licorice root has been used by doctors for successive generations for the treatment of abdominal pain, diarrhea, pus, blood stool, and other gastrointestinal symptoms [4]. The main components of HQT were baicalin, wogonoside, paeoniflorin, glycyrrhizic acid, mucorin, and A-7-O-glucuronide by high-performance liquid chromatography (HPLC) [5, 6]. Baicalin can inhibit NF-KB and regulate Treg/Th17 cell balance in the colon tissue of experimental animals so as to exert anti-inflammatory and immunomodulatory effects [7, 8]. Wogonin, paeoniflorin, and glycyrrhetinic acid can upregulate the tight junction proteins such as claudin-1 and zonula occluden-1 (ZO-1), thereby alleviating intestinal barrier function impairment [6]. HQT has been effectively used to treat ulcerative colitis in clinic [9]. In addition, HQT can effectively alleviate experimental ulcerative colitis induced by dextran sulfate sodium (DSS) and 2,4,6-trinitrobenzene sulfonic acid (TNBS) [10, 11]. Animal studies show that HQT can inhibit the pathway (RasPI3K-Akt-HIF-1a and NF-kB) in colon tissue from playing an anti-inflammatory role [12]. It has also been reported that HQT can regulate the balance of Th cell subsets to play an immunomodulatory role [6, 13, 14]. In addition, miRNA abnormalities are considered to be closely related to the occurrence of UC. MiR-192/miR-122/miR-29 can regulate the expression of Toll and NOD-like receptors in intestinal innate immunity cells, leading to the occurrence of UC [15–17]. miR-210/miR-155 can regulate the expression of cytokine IFN-*γ*/transcription factor Hif1a/IL-2, thus affecting the differentiation and function of intestinal adaptive immune cells [18–20]. The reduction of miR-21 and miR-200B can injure the intestinal epithelial barrier function [21–25]. However, whether HQT can effectively regulate miRNA is unclear. In this article, we plan to use colon mucosa tissue to sequence and analyze miRNA and clarify the miRNA that HQT can regulate, so as to provide a scientific basis for improving the therapeutic mechanism of HQT.

## 2. Materials and Methods

### 2.1. Preparation of HQT

According to the method reported in our literature, raw medicinal materials including Radix *Scutellariae*, Radix *Paeoniae Alba*, *Glycyrrhizae* Radix, and Fructus Jujubae were mixed according to the ratio of 9 : 6:6 : 49 [12]. The raw medicinal materials were soaked in 10 times the volume of distilled water for 30 min, and decocted them at 100°C for 30 min. Then, the extract was collected from the decocted water. 10 times the volume of distilled water was added to the herb residue, and a decoction for it of 30 min was needed. The extract needed to be collected, and the two extractive solutions needed to be combined, concentrated, and freeze-dried.

### 2.2. Animal Modeling

Twenty-four male Balb/c mice purchased from Guangdong Medical Laboratory Animal Center (certificate No: SYXK (Guangdong) 2018-0002) were randomly divided into 3 groups, with 8 mice in each group. The groups were named the control group (distilled water), the DSS group (3%DSS + distilled water), and the HQT group (3%DSS + 4.55 g/kg HQT) [12]. Except for the normal group, mice in other groups were free to drink 3% DSS solution. Meanwhile, mice in the HQT group were given a gavage of 0.1 mL/10 g HQT every day [26], and mice in the normal group and model group were given a gavage of 0.1 mL/10 g distilled water. The model was administered continuously for 7 days, during which the activity state, mental state, and hair gloss of mice were observed every day, and the diet and water consumption, fecal characteristics, blood stool, and body mass were recorded, and a disease activity index (DAI) score was made. On the eighth day of the experiment, the mice in each group were killed and the length of their colon tissue was recorded. Observe whether there are edema, adhesion, ulcer, necrosis, and other pathological changes on the intestinal lumen side of the colon, and a pathological score needs to be given. In addition, the colon tissues were divided into three parts for the subsequent WB test, RT-PCR test, and hematoxylin-eosin staining (HE). The study was approved by the local ethics committee of Guangdong Medical University under the No. GT-IACUC201909121.

### 2.3. miRNA Sequencing Analysis

Total RNA was extracted from colon tissue. Firstly, the integrity of RNA was tested by the Agilent 2100 Bioanalyzer. The TruSeq Small RNA Sample prep Kit was used to construct a small RNA library, and the library was amplified and enriched by PCR. Then, the purified library was selected by gel electrophoresis, and the library quality was inspected by Agilent High Sensitivity DNA Kit. The qualified library should have a single peak and without adaptor. The library was further quantified by Quant-iT PicoGreen dsDNA Assay Kit. Finally, sequencing was performed on the Illumina platform. Data analysis includes the following: (1) Based on the reference genome, conduct adaptor removal and quality filtration for the data, annotate the small RNA sequence which is removed from the duplicated sequence, and annotate the abundance. (2) Focus on analyzing the characteristics and expression quantity of miRNA. (3) Carry out cluster analysis and target gene prediction of differential miRNA. Analyzed by DESeq (Version 1.18.0, Anders S and Huber W, 2010), the miRNA met the requirement of (|log_2_FoldChange| > 1) and *P* < 0.05 was defined as a differential miRNA. The miRNA sequencing analysis was completed by Nanjing Personal Gene Technology Co., Ltd.

### 2.4. RT-PCR

In this experiment, the TB green chimeric fluorescence method was used for test, and the operation was carried out according to the instructions of the Takara real-time fluorescence quantitative kit. The mice miRNA-185-3p primers were purchased from Guangzhou Ribo Biotechnology Co., Ltd. (Mira10011661-100). *β*-actin: F: CTTCTTTGCAGCTCCTTCGTT, R: AGGAGTCCTTCTGACCCATTC. PCR reaction system: 12.5 *μ*L TB Green Premix Ex Taq II (2X), 1 *μ*L PCR forward primer (10 *μ*M), 1 *μ*L mRQ 3′ primer (10 *μ*M), 2 *μ*L DNA template (<100 ng), 8.5 *μ*L DEPC water, and total volume 20 *μ*L.

PCR reaction condition: hold (1 cycle) 95°C, 30 s; PCR (40 cycle) 95°C, 5 s, 60°C, 30 s; dissociation (1cycle) 95°C, 15 s, 60°C, 30 s; 95°C, 15 s. The average value of the three tubes was taken and the relative value of miRNA-185-3p/*β*-actin was calculated.

### 2.5. Western Blotting

Adding protease inhibitor and phosphatase inhibitor (Solarbio, BC3711) into the lysate to fully extract protein, centrifuging at 4°C and 12000 rpm for 30 min to obtain supernatant. The protein concentration was tested by the bicinchoninic acid (BCA) method, and 20 µg protein of loading was applied for electrophoresis, followed by PVDF membrane transfer for 1 h, 5% skimmed milk powder sealing for 1 h, and primary antibody incubation at 4 degrees Celsius overnight: B-actin (Affinity AF7018, 1 : 10000), p-MLC (Affinity AF3829, 1 : 200), MLC (Affinity AF8618, 1 : 500), MLCK (Affinity AF5314, 1 : 500), and occludin (Invitrogen 71-1500, 1 : 1000). Add the secondary antibody of the corresponding species and incubate it for 1 h, then conduct ECL development, and finally use Image J software for grayscale analysis.

### 2.6. CaCo_2_ Cell Barrier Injury Model

The logarithmic growth phase CaCo_2_ cells were digested into cell suspension, which needed to be planked. Cultured the cell suspension for about 14 days to grow it into dense monolayer epithelial cells, and the TEER value (ion current resistance, which is related to the integrity of the tight junction between cells) was tested. According to the literature report, the TNF-*α* concentration of 10 ng/mL was used [27], and the change rate of the TEER resistance value was tested after 24 hours = TEER value before treatment/TEER value after treatment. According to the experimental design, the control group, the TNF-a group, and the HQT group (TNF-*α* + HQT50 *μ*g/mL) (HQT dose was confirmed by concentration gradient) were set. Each group had 3 parallel holes. After continuing to put the cell suspension in the incubator for 24 hours, the change rate of TEER was tested, and samples were collected to extract RNA and protein.

### 2.7. Cell Transfection

According to the instruction manual of RNAi products of GenePharma, the experiment set for the inhibitor NC group, the inhibitor miRNA-185-3p group, the mimic NC group, and the mimic miRNA-185-3p group, and the synthetic sequences were as follows: inhibitor NC: 5′-CAGUACUUUUGUGUAGUACAA-3′, inhibitor miRNA-185-3p: 5′-GACCAGAGGAAAGCCAGCCCCU-3′, mimic NC: 5′-UUGUACUACACAAAAGUACUG-3′, and mimic miRNA-185-3p: 5′-AGGGGCUGGCUUUCCUCUGGUC-3′. Planking CaCo_2_ cells. Diluting lip-3000 and oligo with Opti-MEM, respectively, mixing them at a ratio of 1 : 1. Make the solution standing and adding CaCo_2_ cells into it, testing TEER value and collecting samples after transfection for 72 h.

### 2.8. Statistical Analysis of Data

Adopting SPSS19 statistical software, the single factor analysis of variance method was used for comparison among multiple groups, and a two-side *t*-test was used for comparison between two samples. *P* < 0.05 was defined as statistically significant.

## 3. Results

### 3.1. Protective Effect of HQT on the UC Barrier Function Injury Model

**Figure 1:**
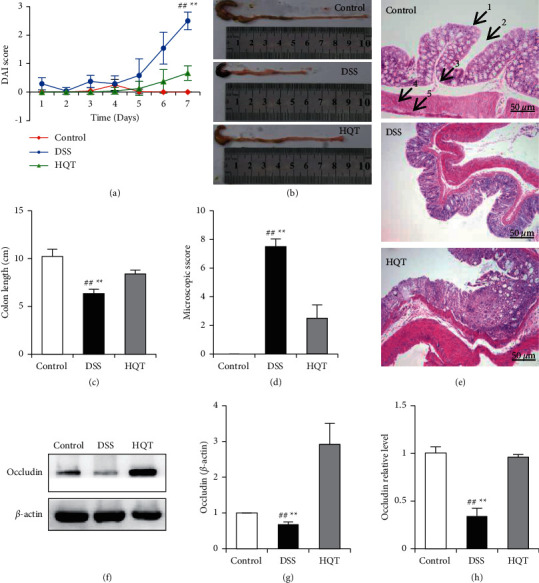
Therapeutic effect of HQT on barrier function injury of DSS-induced ulcerative colitis (UC) mice. Experimental mice were given a gavage of 3% DSS for modeling for 7 days, and the mice in the HQT group were given a gavage of 4.55 g/kg HQT for treatment for 7 days. DAI score of mice in the control group, the DSS model group, and HQT (DSS model group plus HQT treatment) group (a); colon length and its statistics (b, c); colon tissue macroscopic score (d); pathological changes of colon were observed by HE staining (e) (200×; 1. villi, 2. crypt, 3. submucosa, 4. muscularis, 5. serosa); the relative expression quantity of colon occludin was tested by WB (f, g) and RT-PCR(H) (*n* = 8; ^#^*P* < 0.05; ^##^*P* < 0.01; ^*∗∗*^*P* < 0.01).

The DAI score is used to evaluate the changes of animal symptoms after DSS modeling, which includes three indexes: weight loss ratio (%), stool characteristics, and blood stool. It can be seen that the DAI score of mice in the HQT group is significantly lower than that of mice in the DSS group (*P* < 0.01) (Figure 1(a)). In the DSS model group, the colon of mice developed a contracture deformity, and the colon length was significantly shortened (*P* < 0.01). After HQT treatment, the colon length of mice was significantly prolonged (*P* < 0.01) (Figures 1(b) and 1(c)). Compared with the control group, the colon tissue of mice in the DSS group showed obvious congestion, edema, adhesion, and ulcer. Mucosal injury, crypt structure disorder, gland deformation, arrangement disorder decreased, goblet cells decreased, submucosa edema, and muscularis edema thickened, and a number of inflammatory cells infiltrated, while HQT significantly improved the pathological changes of the colon (Figure 1(e)). Transcriptional expression of occludin in colon tissue of mice in the DSS group was significantly decreased, and HQT treatment could reverse the downregulation (*P* < 0.01) (Figures 1(f)–1(h)).

### 3.2. Sequencing of miRNA in Colonic Mucosal Tissue

**Figure 2:**
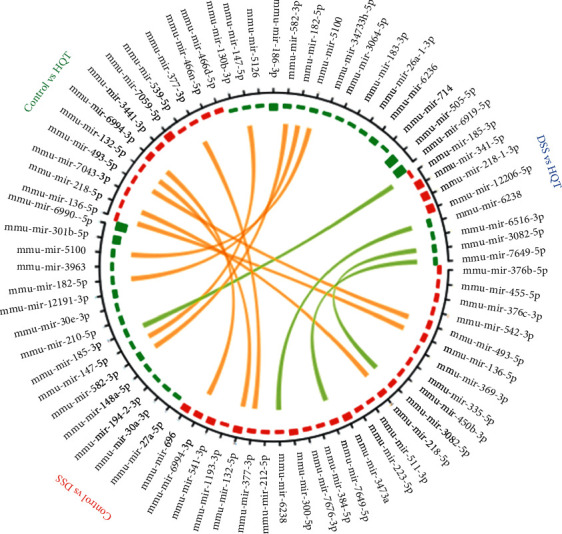
Differential miRNA gene between groups. Range from the outer ring to the inner ring: differential gene names (if the genes are too many, they would not be displayed). The gene express abundance difference: red represents upregulation and green represents downregulation. The connecting lines represent the common differential genes between the two differential analyses.

Genomic miRNA sequencing of mouse colonic mucosal tissue was carried out, which was divided into 3 groups, with 8 samples in each group, and 24 samples in total. The groups are named the control group, the DSS model group, and the DSS + HQT treatment group, respectively. The sequencing data quantity was 20 M/sample, and 1389 genes were found. Differential gene screening should satisfy |log2FoldChange| > 1 and *P* value <0.05. Compared with the control group, 26 genes were upregulated and 15 genes were downregulated in the DSS group. Compared with the DSS model group, the HQT + DSS treatment group had four genes upregulated and four genes downregulated. Compared with the control group, miR-185-3p was significantly downregulated (downregulated by 2.38 times) in the DSS model group; in comparison with the DSS model group, miR-185-3p was significantly upregulated (upregulated by 2.02 times) in the HQT + DSS treatment group. This indicates that miR-185-3p is a potential regulatory gene for HQT treatment (Figure 2). miR-185-3p simultaneously satisfies two conditions: one is meeting differential gene screening requirements; the other is that gene changes can be reversed by HQT treatment, indicating that miR-185-3p is a potential regulatory gene for HQT treatment (Figure 2).

### 3.3. HQT Regulates the Expression of Mirna-185-3p in Colon Tissue

**Figure 3:**
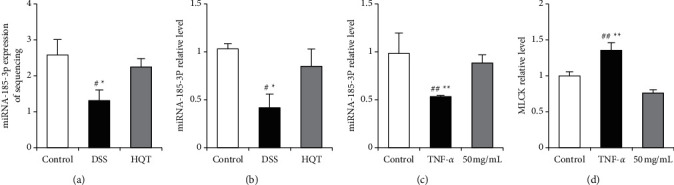
HQT regulates miRNA-185-3p. Experimental mice were given a gavage of 3% DSS for modeling for 7 days, meanwhile the mice in the HQT group were given a gavage of 4.55 g/kg HQT for treatment for 7 days.

Compared with the control group, miRNA-185-3p was significantly downregulated in mice in the DSS animal model, and the downregulation of miRNA-185-3p was reversed after HQT treatment (Figure 3(b)), which was consistent with the results of miRNA sequencing (Figure 3(a)). MLCK is a direct target gene of miRNA-185-3p, which is upregulated in the DSS animal model group, and the upregulation of MLCK can be significantly reversed after HQT treatment (Figure 3(d)). In addition, HQT can also reverse the downregulation of miRNA-185-3p induced by TNF-*α* in the injury model of CaCo_2_ monolayer cell barrier mediated by TNF-*α* (Figure 3(c)). These results indicate that HQT can regulate the expression of miRNA-185-3p.

### 3.4. Expression of miRNA-185-3p That HQT Regulates CaCo_2_ Monolayer Cell Barrier Function

**Figure 4:**
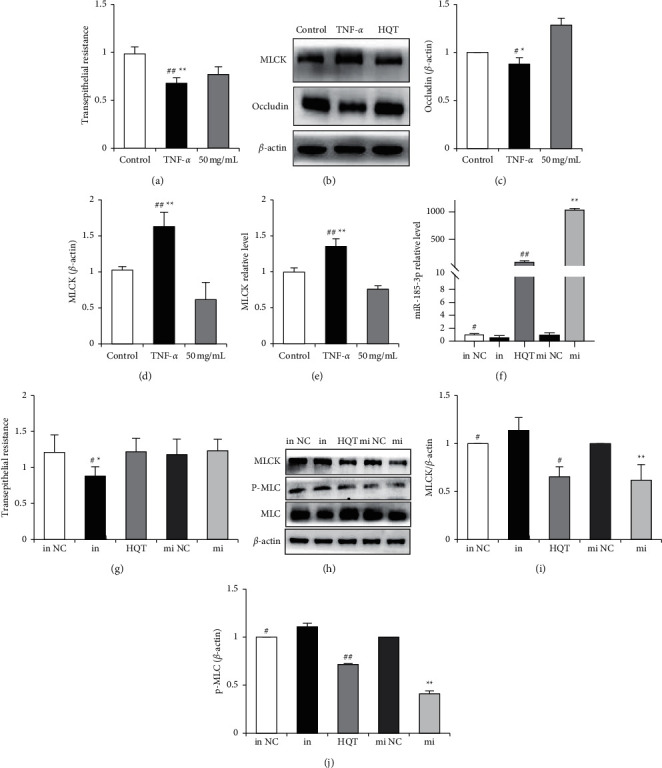
The expression of miRNA-185-3p that the barrier function in CaCo_2_ monolayer cells regulating by HQT. The model group was treated with TNF-*α* for 24 hours after CaCo_2_ cells grew into a monolayer, and the HQT groups were treated with TNF-*α* for 2 hours. Then, 50 *µ*g/mL of HQT was added to the CaCo_2_ cells. After 24 hours, the TEER value was observed (a). The expression of MLCK and occludin protein was tested by WB (b–d). The expression of MLCK mRNA (e) was tested by q-PCR. Mimic NC, mimic miRNA-185-3p (mi), inhibitor NC, and inhibitor miRNA-185-3p (in) were transinfected, respectively, by CaCo_2_ cells. After adding HQT into miRNA-185-3p (f), the change of the TEER value (g) and the change of MLCK/p-MLC (h, i, j), their expression were tested (*n* = 3, ^*∗*^*P* < 0.05, ^*∗∗*^*P* < 0.01; ^#^*P* < 0.05, ^##^*P* < 0.01). *β*-actin.

In the TNF-*α* induced model group, the TEER value decreased by 36.32%, while in the HQT group, it increased by 22.58% compared with that of the TNF-*α* group (*P* < 0.01) (Figure 4(a)). Occludin, a tight junction protein induced by TNF-*α*, decreased significantly, but was recovered by the HQT treatment group (*P* < 0.05) (Figures 4(b) and 4(c)). Meanwhile, HQT could reverse the upregulation of MLCK transcription induced by TNF-*α*(*P* < 0.01) (Figures 4(b), 4(d), and 4(e)). In addition, in CaCo_2_ monolayer cells, firstly, it was proved that overexpression of miRNA-185-3p and interference of miRNA-185-3p expression were effective (Figure 4(f)). Inhibitor miRNA-185-3p expression decreased the TEER value by 0.3361 (*P* < 0.05), while the overexpression of miRNA-185-3p expression increased the TEER value by 0.053 (*P*=0.7482) (Figure 4(g)). Interference of miRNA-185-3p caused the downstream target gene MLCK/p-MLC to be upregulated correspondingly(*P* < 0.05), while overexpressing miRNA-185-3p did just the opposite (*P* < 0.01). Meanwhile, the effect of HQT on regulating the miRNA-185-3p/MLCK/p-MLC signaling pathway is similar to that of overexpressing miRNA-185-3p (Figures 4(h)–4(j)).

## 4. Discussion

The mouse acute colitis model induced by DSS is the UC closest to human beings. It can injure the colon epithelial barrier function, with the characteristic of being simple, rapid, and repeatable. The injury of the colon mucosa can lead to a decrease of the expression of intestinal mucosal tight junction proteins such as occludin and ZO-1. The exposure of intestinal small vessels and the decrease of intestinal reabsorption, result in bloody stool, loose stool, and weight loss [28]. TNF-a activates the ATPase at the head of the actin heavy chain, and the energy generated causes the cytoskeletal actin microfilament to slide and the actin ring to contract, which ultimately destroys the normal distribution of tightly attached proteins, thereby increasing intestinal epithelial permeability. Therefore, TNF-a is often used to establish the barrier injury model of CaCo_2_ monolayer epithelial cells [29, 30]. After UC mice were given a gavage of dextran coupled with fluorescein isothiocyanate (FITC) as a tracer to reflect intestinal epithelial permeability, Scholar Zou et al. found that HQT could significantly reduce FITC content in the blood of mice. This study further confirmed that immunohistochemical results of colon tissues showed that HQT could upregulate the expression of ZO-1 and occludin [11], which indicates that HQT has a protective effect on the intestinal epithelial barrier function of mice. In this study, a model of intestinal epithelial barrier injury in vitro and in vivo induced by DSS and TNF-a was used to simulate the pathological changes of the intestinal mucosal barrier during the occurrence of UC and further confirm that HQT has the function of protecting the intestinal epithelial barrier, which is consistent with the results of previous studies.

In the past, the research on the therapeutic mechanisms of HQT focused on regulating immunity, inhibiting inflammation, and regulating intestinal flora. HQT can inhibit the expression of NF-*κ*B, JAK/STAT, MAPK, and other inflammatory pathways in colon tissues or cells of experimental mice [11, 12, 26, 31, 32]. HQT can inhibit the expression of Th1/Th17-related transcription factors such as T-bet and ROR-*γ*t and upregulate the expression of Th2/Treg-related transcription factors GATA-3 and Foxp3, thus regulating the balance of CD4+ T cell subsets and the secretion of corresponding cytokines [13]. In addition, HQT can reverse the imbalance of intestinal flora, promote the quantity growth of probiotics and lactobacillus, and inhibit the proliferation of *Vibrio* desulphurizer and *Clostridium tenella* [12]. In this study, we focused on the mechanism of regulation of HQT on miRNA and found for the first time that HQT can regulate miR-185-3p.

**Figure 5:**
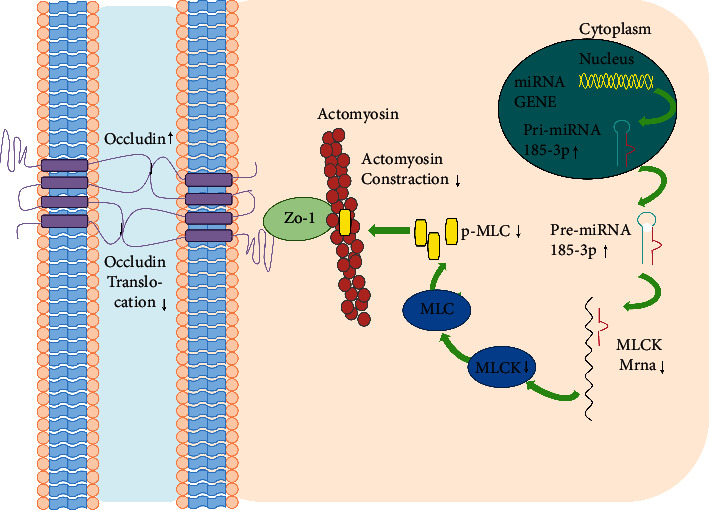
Pattern of regulating the intestinal epithelial barrier by HQT. HQT upregulated miRNA-185-3p expression, thereby inhibiting MLCK expression, further decreasing p-MLC protein and ultimately decreasing cytoskeleton protein contraction, inhibiting cell membrane occludin protein translocation, and promoting increased occludin protein expression.

Scholar Dan Ma used a TNF-a-induced barrier injury model of CaCo_2_ monolayer cells, and found that the inhibitor miR-185-3p can damage barrier function. On the contrary, upregulating miR-185-3p can enhance barrier function, indicating that miR-185-3p can maintain barrier function. Furthermore, the target gene of miR-185-3p was verified to be MLCK by a dual-luciferase reporter gene experiment, and then it was confirmed that MLCK could upregulate p-MLC (T18 and S19), thereby activating ATPase at the head of the actin heavy chain, destroying the normal distribution and expression of tight junction protein and leading to the increase of intestinal epithelial permeability [29, 33]. The increase of MLCK expression was observed in colon tissue of IBD patients, which was positively correlated with the degree of pathological changes of the tissues [34]. When mice express continuously activated MLCK, it can increase the expression of p-MLC as well as intestinal epithelial permeability, which can be reversed by an MLCK inhibitor [35–38]. Scholar Huang et al. found that Pulsatilla decoction can regulate tight junction protein through the MLCK/p-MLC pathway to improve intestinal epithelial barrier function in UC mice [39]. Scholar Du et al. found that adrenomedullin, 1,25-(OH)2-VitD3, and other active molecules can reduce the expression of MLCK/p-MLC, and then reduce intestinal epithelial permeability and relieve UC [40, 41]. Therefore, regulating the distribution of tight junction protein by intervening in the MLCK/p-MLC pathway can be regarded as a breakthrough point to improve the intestinal epithelial barrier. In this study, HQT significantly alleviated the injury of the CaCo_2_ monolayer cell barrier caused by TNF-a, which not only increased the expression of occludin, a tight junction protein but also upregulated the expression of miR-185-3p and downregulated MLCK/p-MLC, indicating that HQT can take effect by regulating the miR-185-3p/MLCK/p-MLC pathway. Furthermore, using the DSS-induced UC model of mice, it was found that HQT could also upregulate the expression of miR-185-3p and tight junction protein occludin. The abovementioned content indicated that HQT could mediate the miR-185-3p pathway to intervene in the occurrence of UC (Figure 5).

For analysis of miRNA differential gene sequenced in colon mucosa and GO functional enrichment analysis, it mainly involves protein modification processes (GO: 0036211) and macromolecular modification (GO: 0043412). The modification can regulate the localization, metabolism, function, degradation, and activity of proteins. In addition, cellular protein localization (GO: 0008104), macromolecular localization (GO: 0033036), signal regulation (GO: 0023051), and localization regulation (GO: 0032879) were also enriched. It is speculated that many proteins, such as tight junction proteins, are redistributed during UC, which leads to the impairment of intestinal epithelial barrier function. Then, KEGG analysis showed that the enrichment pathway involved cytoskeleton regulation pathway (Mmu04810), adhesion junction pathway (Mmu04520), and bacterial invasion pathway (Mmu05100), which were closely related to intestinal epithelial barrier function. At the same time, immune regulation pathways, such as the B cell receptor pathway (Mmu04662), T cell receptor pathway (Mmu04660), and Fc-*γ* receptor-mediated phagocytosis pathway (Mmu04666), were also enriched, suggesting that UC is an autoimmune disease. In addition, the enrichment of the MAPK signaling pathway (Mmu04010) and autophagy pathway (Mmu04140) suggested that inflammation and autophagy were involved in the occurrence of UC. Finally, using KO analysis, we observed that miRNA target genes participated in the regulation of the MLCK pathway, MAPK pathway, and T cell receptor pathway, among which MLCK was considered as a miR-185-3p target gene, which is one of the reasons why this paper focuses on the miR-185-3p study. However, we focused on finding the differential miRNA regulated by HQT, but unfortunately only miR-185-3p was found, which is supposed to be the reason for the small amount of sample data (20 M).

## 5. Conclusion

In this study, for the first time, we observed that HOQ could protect UC barrier function by upregulating miR-185-3p, then inhibiting the MLCK/p-MLC pathway, and finally interfering with occludin through the mouse UC model induced by DSS combined with the injured CaCo_2_ monolayer cell barrier induced by TNF-a.

## Data Availability

The data used to support the findings of this study are included within the article.
